# COVID-19 Infection Among Healthcare Workers at a Tertiary Healthcare Hospital

**DOI:** 10.7759/cureus.43412

**Published:** 2023-08-13

**Authors:** Hamna Abdul Muthalib, Alaa Hamad, Safwan Abbasi, Faaezuddin Syed, Hanan S Alamer, Esam Albanyan, Suliman Aljumaah, Salem AlGhamdi

**Affiliations:** 1 Medicine, Alfaisal University College of Medicine, Riyadh, SAU; 2 General Medicine, Alfaisal University College of Medicine, Riyadh, SAU; 3 Pediatric Infectious Diseases, King Faisal Specialist Hospital and Research Centre, Riyadh, SAU

**Keywords:** pandemic, healthcare providers, infection, healthcare, saudi arabia, covid-19

## Abstract

Purpose: SARS-CoV-2 or COVID-19 virus was the culprit of the global pandemic that began in 2019. With alarming mortality rates reaching sky-high worldwide, the virus prompted the masses to switch to online working. However, this was not feasible for healthcare workers (HCWs) exposed to a higher-than-normal risk of acquiring COVID-19 infection. This study aims to observe the prevalence of COVID-19 positivity among the various areas of a healthcare facility in Saudi Arabia.

Methods: A cross-sectional study of positive employees among all departments at a tertiary care hospital in Riyadh, Saudi Arabia, such as administration, capital projects/facilities, and healthcare. The study included all hospital employees-permanent staff, rotating physicians, and trainees-who tested positive for COVID-19 between March 20, 2020 and December 30, 2020.

Results: It was found that HCWs had the most significant number of infected individuals with nursing staff being the predominant demographic. This was followed by the capital projects/facilities departments, of which the environmental services staff were the most infected.

Conclusion: It is pertinent that strict protocols be taken by hospital management to limit the spread of future infectious diseases within hospital settings. This includes the provision of personal protective equipment (PPE) and adequate education on its proper usage, alongside regular surveillance of staff with regard to adherence and early detection of symptoms.

## Introduction

SARS-CoV-2 or COVID-19 virus belongs to a family of viruses known as coronavirus. With a varying onset and presentation of symptoms, including no symptoms at all, SARS-CoV-2 was responsible for the pandemic in 2019 [1]. With a mortality rate ranging from 3% to 6%, the virus has especially shown disastrous effects on patients with underlying comorbidities. Patients admitted to the intensive care unit, the elderly, and the immunocompromised, to name a few, are especially prone to a more serious infection [2].

The rapidly spreading infection forced the ruling authorities of most countries to implement quarantine, isolation, and contact-tracing protocols [3]. The main motivation for implementing these measures was to combat the contagious spread and prevent its destructive outcomes [4]. While the majority of the work was detoured to the online platform, professionals in health care were exposed to higher than-usual working hours in the hospital. The exponential spread of infection required healthcare workers (HCWs) to upscale their working hours, posing serious threats to their mental and physical health. They were more susceptible to the infection depending on the varying degrees of exposure, with studies reporting between 3%-17% infection rate in HCWs [5]. A staggering statistic reported in Italy showed over twelve thousand infected and over a hundred HCWs dead as a result of COVID-19. Even though these numbers were recorded as of April 2020, figures continued to rise [6].

The lack of proper personal protective equipment (PPE) supply or its adherence, among many other factors, rendered HCWs especially vulnerable to the virus [7]. Our objective of this study is to determine the prevalence of COVID-19 infection among healthcare staff in a tertiary hospital in Saudi Arabia.

## Materials and methods

This is a cross-sectional study, that took place in King Faisal Specialist Hospital and Research Center (KFSHRC), Saudi Arabia. KFSHRC is a tertiary care hospital that offers a wide range of medical services, education, and research activities with a total of around 12,400 staff. We have defined HCWs in this study as all workers that work within the hospital setting as they are all potentially implicated, whether directly or indirectly, by hospital-acquired infections such as COVID-19. The majority of staff are working under healthcare delivery such as medical departments and patient flow management. The remaining group is mainly administrative workers concerned with jobs related to financing, IT services, recruiting, risk management, maintenance, food services, drivers, research, and training. In this study, staff was classified into three main categories according to their potential contact with patients-which includes; Health care, Capital project and facilities, and Administration.

Included in the study were all hospital employees-permanent staff, rotating physicians, and trainees who tested positive for the COVID-19 polymerase chain reaction (PCR) from March 20, 2020 to December 30, 2020, whether during surveillance or based on clinical presentations.

Since the pandemic started, all symptomatic employees were required to be off work for five days and do COVID-19 tests. Exposed staff were asked to fill out a contact tracing form that allocate them to a category of risk. If the intended staff was categorized as low risk, they need to be screened for COVID-19 after 24 hrs from last exposure. If they were categorized as High risk, they need to be off work for five days and screened for COVID-19 after five days from last exposure. The travel screening clinic also required employees returning from abroad to do COVID-19 tests on their return.

HCW data was obtained from the Infection Control and Hospital Epidemiology Department and included age, sex, medical department, date of positive COVID-19 PCR, symptoms status, past medical history, and source of infection. Prevalence is reported using frequency distribution.

## Results

A total of 1,763 individuals tested positive for SARS-CoV 2 from March 20, 2020 to December 30, 2020, with an age range of 29 to 49 years old and a mean age of 39. Males accounted for 60% of those who tested positive, while the rest were females. Patient demographics and characteristics are detailed in Table 1.

**Table 1:** Sample characteristics (Total = 1,763)

Characteristics	n (%)
Age (Mean ± SD )	39 ± 10 years
Sex	
Female	712 (40)
Male	1,051 (60)
Symptoms	
No	792 (45)
Yes	904 (51)
Missing	67 (4)
Risk factors	
No	1,414 (80)
Diabetes	67 (4)
Hypertension	104 (6)
Asthma	19 (1)
Pregnancy	20 (1)
Other	5 (<1)
Missing	99 (6)
Source of infection	
Hospital-associated	266 (15)
Community-associated	289 (16)
Missing	1,208 (69)

**Figure 1:**
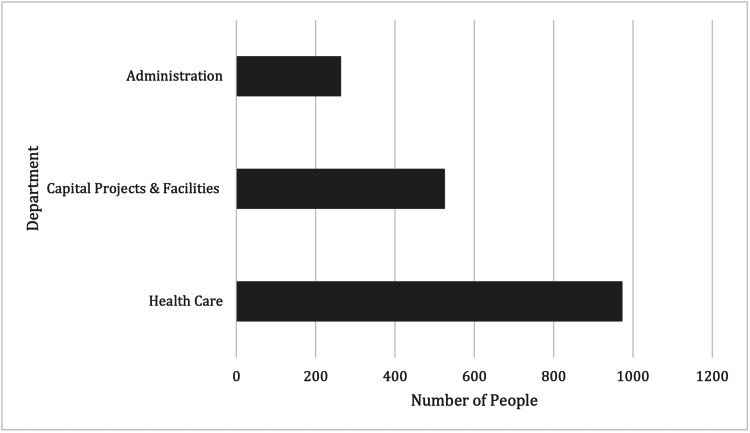
Number of people testing positive for SARS-CoV-2 according to department

The subjects belonged to various departments at the hospital. The majority were from healthcare divisions at 55% (Figure 1).

**Figure 2:**
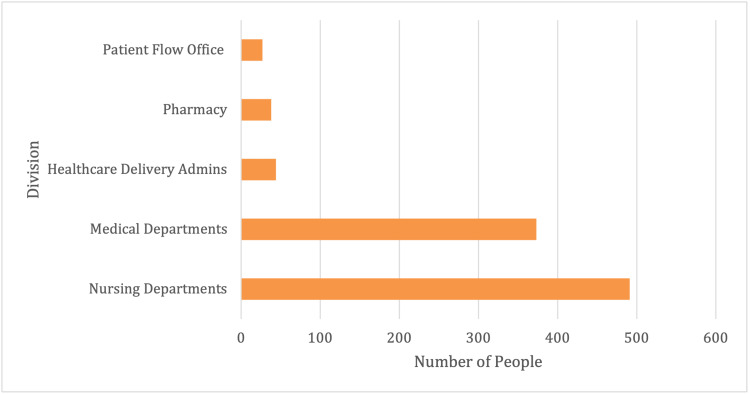
Healthcare department and SARS-CoV-2 positivity by division

Of the 973 individuals who tested positive and belonged to the healthcare division, the majority were employees in the nursing department (50%), while 38% were from the medical departments, and the remaining included pharmacy, admin, and patient flow staff (Figure 2). 

**Figure 3:**
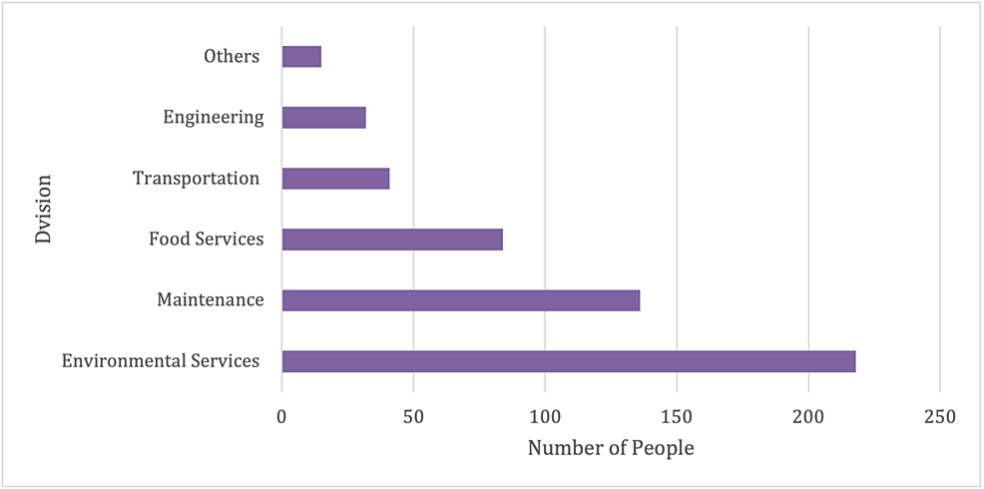
Capital projects and facilities department and SARS-CoV-2 positivity by division

Out of the 1,763 subjects who tested positive, 30% were employed in capital projects and facilities (Figure 3).

**Figure 4:**
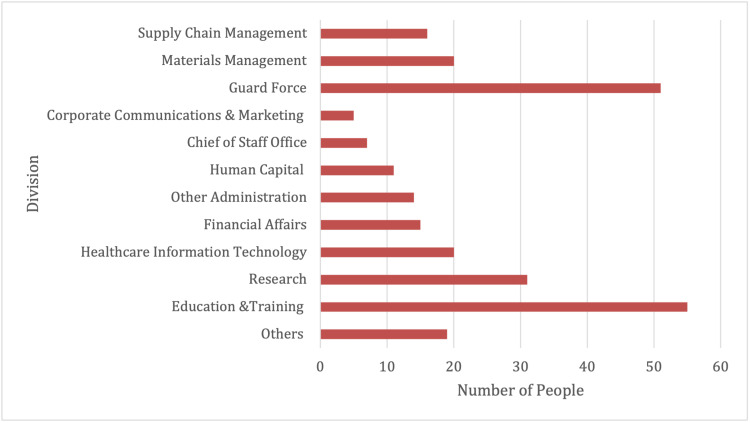
Administrative departments and SARS-CoV-2 positivity by division

Of these, most of the individuals were part of environmental services (41%), maintenance (26%), and food services (16%). Executive officers who tested positive accounted for 6% of the study population, with guard forces comprising the majority (52%), while materials and supply chain management staff among others accounting for the rest (Figure 4).

Other departments whose employees tested positive encompassed education and training, research, information technology, financial affairs, and administration. 

The symptoms and a few risk factors related to COVID-19 infection, and its severity were also assessed. Of the 1763 people testing positive, 904 (51%) were symptomatic and 792 (45%) were asymptomatic. 250 (14%) subjects had risk factors predisposing to COVID-19 infection and its severity, such as hypertension (6%), diabetes (4%), hypertension and diabetes (2%), asthma (1%), and pregnancy (1%). 

The source of infection acquirement was also assessed. For most of the study subjects, information regarding this was missing and for those available, hospital sources and community sources accounted for similar proportions.

## Discussion

This study aimed to investigate the prevalence of COVID-19 infection among the HCWs of a tertiary care hospital. We found that medical professionals directly in contact with patients from the healthcare department were the most affected by the spread of COVID-19. Among this department, the nursing the medical sector were the most susceptible and were the victims of the spreading infection. This finding reflects the trend observed worldwide, including Italy, the United States, China, etc., with higher infection rates prevalent among direct providers of patient care as opposed to other departments in the hospital [5,8-10].

Surprisingly, higher infection rates were also discovered among the personnel employed in the capital projects and facilities department. Environmental services, maintenance, and food services individuals were the highest to be affected in this division. This can be attributed to either exposure to undetected infection prevalent among different patients and coworkers or issues related to PPE. One of the major factors concerning PPE usage predisposing hospital staff to COVID-19 infection is the lack of adequate access to it at times of utmost need. Other factors have been reported as well, such as deficiency in the information received about the use of PPE, inability to apply proper techniques for the donning and doffing by the workers, and scarcity of COVID-19 testing kits during the initial phase of the pandemic [11,12].

The symptoms of patients infected with COVID-19 were also assessed by this study. Even though a majority of the care providers displayed symptoms of infection (51%), a large proportion of the study population was asymptomatic (45%). These individuals might have been unaware of the infection and possibly be a vital source of its spread. Serological testing of the individuals exposed to COVID-19 patients should have been more frequently utilized to identify these asymptomatic or subclinical infections in the healthcare setting [13].

Even though a majority of the study population were not able to identify the source of infection, of those who recognized, near equal distribution of infection acquired in hospital and community were reported. The higher prevalence of hospital-acquired COVID-19 infection is of particular concern. Several studies, including ours, have described and presented the remarkable COVID-19 infectivity rates among hospital staff despite several employed protective measures [14,15]. In order to curb the spread of infection, strict protocols should be deployed by the hospital management with regard to the use of PPE, periodic surveillance of the hospital staff with proper diagnostic tools, and adequate working conditions for the providers are essential to combat the spread of infection.

## Conclusions

In conclusion, this research study focused on the prevalence of COVID-19 infection among HCWs in a tertiary care hospital. The findings revealed that medical professionals within the healthcare department, particularly nursing and medical sectors, were the most affected by the spread of COVID-19, consistent with global trends observed in other countries. Higher infection rates were also identified among personnel employed in the capital projects and facilities department, indicating potential issues with PPE access and usage. The study highlighted the significance of proper PPE usage and training for HCWs, as well as the need for periodic surveillance and serological testing to identify asymptomatic carriers within the healthcare setting. Stricter protocols and adequate working conditions are crucial for reducing the spread of infection in hospitals. Addressing these factors will be essential to safeguard the health and well-being of healthcare providers and ultimately contribute to controlling the spread of COVID-19 in healthcare facilities.
